# Emergency hospital services utilization in Lleida (Spain): A cross-sectional study of immigrant and Spanish-born populations

**DOI:** 10.1186/1472-6963-8-81

**Published:** 2008-04-10

**Authors:** Montserrat Rué, Xavier Cabré, Jorge Soler-González, Anna Bosch, Mercè Almirall, Maria Catalina Serna

**Affiliations:** 1 Biomedical Research Institut, Lleida (IRBLLEIDA), Spain; 2 University of Lleida, Lleida, Spain; 3 Hospital Universitari Arnau de Vilanova, Lleida, Spain; 4 Rambla de Ferran Health Center, Institut Català de la Salut, Lleida, Spain; 5 Regional Primary Care Management Office, Institut Català de la Salut, Lleida, Spain

## Abstract

**Background:**

The use of emergency hospital services (EHS) has increased steadily in Spain in the last decade while the number of immigrants has increased dramatically. Studies show that immigrants use EHS differently than native-born individuals, and this work investigates demographics, diagnoses and utilization rates of EHS in Lleida (Spain).

**Methods:**

Cross-sectional study of all the 96,916 EHS visits by patients 15 to 64 years old, attended during the years 2004 and 2005 in a public teaching hospital. Demographic data, diagnoses of the EHS visits, frequency of hospital admissions, mortality and diagnoses at hospital discharge were obtained. Utilization rates were estimated by group of origin. Poisson regression was used to estimate the rate ratios of being visited in the EHS with respect to the Spanish-born population.

**Results:**

Immigrants from low-income countries use EHS services more than the Spanish-born population. Differences in utilization patterns are particularly marked for Maghrebi men and women and sub-Saharan women. Immigrant males are at lower risk of being admitted to the hospital, as compared with Spanish-born males. On the other hand, immigrant women are at higher risk of being admitted. After excluding the visits with gynecologic and obstetric diagnoses, women from sub-Saharan Africa and the Maghreb are still at a higher risk of being admitted than their Spanish-born counterparts.

**Conclusion:**

In Lleida (Spain), immigrants use more EHS than the Spanish born population. Future research should indicate whether the same pattern is found in other areas of Spain and whether EHS use is attributable to health needs, barriers to access to the primary care services or similarities in the way immigrants access health care in their countries of origin.

## Background

The number of immigrants in Spain in the last decade has increased dramatically. In 1996 the immigrant population represented 1.4% of the resident Spanish population, and in 2006 it represented 10.8% [1]. In 2003, more than 2/3 of the immigrant population had been living in Spain for less than 5 years [2]. At this point there are few studies that have evaluated the impact of this migratory phenomenon on our country's health care system and, in particular, on the use of emergency hospital services (EHS).

In Spain, there is a National Health System (NHS), financed mainly by taxes, which provides universal and free health coverage including primary, specialized and hospital health care [3]. Immigrants may register in their municipality of residence to have access to health care, regardless of their legal status [4]. However, a number of immigrants are not registered because they are unaware of the process, fear legal authorities' access to the database, or their municipal government has rejected their registration [5]. All people in Spain, regardless of origin and legal status have the right to emergency care.

It has been estimated that more than half of the population use EHS at least once every year and visits to EHS in Spain in the last decade have increased at a rate of 4% per year [6]. The increase in EHS visits in Spain has been attributed to several factors, among which the following stand out: population increase, partially due to increased immigration; the aging of the population with an increase in chronic diseases; and especially the use of emergency services as an alternative to ambulatory care for non-emergency problems [6-8]. Vazquez Quiroga *et al *[9] found that most users of EHS use them as "on-demand" primary care, a rapid way of obtaining health care. Among reasons for attendance at EHS in a region of Spain were: ignorance of non-hospital emergency services, better clinical resources, quicker care, sensation of vital urgency and poor quality of care in primary care.

There is some evidence that immigrants in Spain tend to use EHS in preference to other health services [10,11]. Circumstances that may explain differential usage of EHS have been described elsewhere. The causes of these differences are not only different lifestyles but also cultural and language barriers, precarious work situations that make it necessary for patients to seek health care services outside of specific hours, as well as immigrants' irregular legal status limiting access to the primary health-care system [12-14].

This study compared the demographics of immigrants and Spanish-born individuals that used EHS. The two groups were also compared based on: EHS diagnoses, EHS utilization rates by zone of origin, mortality, frequency of hospital admission and diagnoses at hospital discharge.

## Methods

### Design

Cross-sectional study in the EHS of a public hospital in the city of Lleida (Spain).

### Setting

Lleida is a city located in northeast Spain, in the autonomic region of Catalonia, with approximately 130,000 inhabitants. Immigrants living in the area are primarily economic immigrants, whose countries of origin have much lower income per capita than Spain. Immigrants represented 17% of the total population of Lleida in December 2006, whereas in all of Catalonia immigrants represented 12.6%. Immigrants living in Lleida are mostly men (approximately 60% versus 40% of women) and younger, on average, than the Spanish-born population. They come mostly from Latin America, the Maghreb, sub-Saharan Africa and Eastern Europe. African immigrants have less education, most have only primary education or non at all. In contrast, Latin American and Eastern European immigrants are better educated, on the average, than the Spanish population. The grand majority of immigrants work in unskilled jobs, followed by service and construction workers [2]. More than 50% of immigrant women work in domestic services and hotel and restaurant industry [15]. Women from Magreb and Subsaharian Africa are mostly housewives and mothers.

The Hospital Universitari Arnau de Vilanova (HUAV) is a public teaching hospital that serves an area with 340,000 inhabitants – the city of Lleida and the south of Lleida province. The HUAV is the only public hospital with EHS in the area. Therefore their EHS are used by most of the population living in Lleida – 97% of all the EHS visits in the catchment area. From January 1, 2004 to December 31, 2005, the HUAV had 168,111 EHS visits.

### Study sample

A total of 96,916 EHS visits from individuals 15 to 64 years old to HUAV during 2004 and 2005 were included in the analysis. They represent 57.7% of all EHS visits.

Children aged 0–14 years (20%) were not included because the immigrant status was registered differently in hospital data files and in the population registry. On the other hand, adults over 65 years old (22.5% of the total number of visits) were excluded in order to improve the comparability of the immigrant and native-born groups. In the immigrant group only 1.6% of the hospital visits were by patients over 65, whereas in the Spanish born population 25.9% of the EHS visits were by patients over 65.

The study was approved by the ethics committee of the HUAV.

### Variables

EHS data were obtained from the HUAV information system. The following data were collected on each visit: demographic characteristics, country of origin, initial diagnosis and destination at discharge (admission, home, etc.). Patients that were admitted to the hospital had additional information collected: hospital diagnoses, length of stay and destination at discharge. The study population was classified into the following groups based on their country of birth: Spain, the Maghreb, Latin America, Eastern Europe, sub-Saharan Africa, other low-income countries and high-income countries. The high-income countries were: EU member states, Switzerland, Finland, the USA, Canada, Japan, Australia and New Zealand. The other low-income countries included in this study were: Bangladesh, Bhutan, Philippines, India, Iraq, Macao, Pakistan, China and Mongolia. These countries were included because one or more of their citizens either use EHS or were registered in the local census.

When Spanish-born patients were compared with immigrants as a whole, patients born in high-income countries were excluded.

### Statistical analysis

We performed a descriptive analysis of patients' demographic characteristics, diagnoses, admission to the hospital and destination at discharge by country of origin.

Multivariate logistic regression was used to estimate odds ratios of hospital admission by country of origin, adjusting for age.

For patients who were residents of Lleida city, utilization rates were calculated by group of origin as the number of visits from a defined group divided by the total residents from that group at the midpoint of the study period. Utilization rates are expressed as visits per 100 inhabitant-years. Ninety-five percent confidence intervals (95% CI) for utilization rates or mortality rates were obtained using the Poisson distribution. Poisson regression was used to estimate the rate ratios and 95% CI of being visited in the EHS with respect to the Spanish-born population, taking into account the age distribution of each group.

In both, logistic and Poisson regression, age was included in the models grouped into 10-year categories.

## Results

### Demographic characteristics of emergency room visits

From January 1, 2004 to December 31, 2005, there were 96,916 EHS visits by 15–64 year old people to the HUAV, 46,904 from residents of Lleida city. Table 1 presents demographic characteristics by immigrant status. Immigrant emergency room visits were predominantly patients from the Maghreb (7.4% of total visits, 34.7% of immigrant visits) followed by Latin America (4.6% of total, 21.4% of immigrants), Eastern Europe (4.5% of total, 21.0% of immigrants), sub-Saharan Africa (3.3% of total, 15.3% of immigrants) and other low-income countries (0.4% of total, 2.0% of immigrants). Visits by patients from high-income countries represented 1.2% of the total and 5.6% of all immigrant visits. Immigrants that used the emergency department were younger than Spanish-born patients.

**Table 1:** Demographic characteristics of emergency room visits from patients 15–64 years old. Arnau de Vilanova Hospital (Lleida, Spain), 2004–2005. N = 96,916.

	**Immigrants**	**Spanish-born**	**Country of origin not known**
	N (%)	N (%)	N (%)

Gender			
- Male	9,935 (21.1)	34,795 (73.7)	2,456 (5.2)
- Female	10,728 (21.6)	37,408 (75.2)	1,594 (3.2)
Age			
- Mean (SD)	31.4 (9.1)	37.0 (13.9)	36.6 (12.8)
			
Country of origin	20,663 (21.3)	72,203 (74.5)	4,050 (4.2)
			
- Latin American	4,423 (4.6)		
- Eastern Europe	4,329 (4.5)		
- Maghreb	7,178 (7.4)		
- Sub-Saharan Africa	3,163 (3.3)		
- Other low income countries	413 (0.4)		
- High income countries	1,157 (1.2)		
Residents of the city of Lleida	11,565 (24,7)	34,046 (72,6)	1,293 (2.8)

### Diagnosis of emergency visits by immigrant status

In immigrant males from 15 to 64 years old, the most frequent emergency room diagnoses were *non-specific symptoms of the abdomen and pelvis, contusions *and *symptoms involving the respiratory system and other chest symptoms*. In Spanish-born males the most frequent diagnoses were *contusions*, *symptoms involving the respiratory and urinary systems*, *non-specific symptoms of the abdomen and pelvis *and *sprains and strains*.

Females 15 to 44 years old were primarily treated for *normal pregnancy *either in the immigrant or the Spanish-born groups. In the 15–44 years old group the distribution of diagnoses within the gynecologic/obstetric group were very similar in the immigrant and the Spanish-born groups. In fact, within the gynecologic/obstetric group, *normal pregnancy *represented 54.4% of diagnoses in native-born women and 56.0% of diagnoses in immigrant women, *early or threatened labor *amounted to 19% of diagnoses in native born, and 16.9% in immigrants, and *abortion *was 9.2% of diagnoses in native-born and 11.4% in immigrants. In women 15 to 24 years old, the diagnosis of *normal pregnancy *represented 13.7% of the total visits in immigrants versus 6.4% in the Spanish-born group. In females 45–64, the most frequent diagnosis was *symptoms involving the abdomen and pelvis *in both groups.

There were 69 deaths in the 15–64 age group, seven in the immigrant group and 62 in the Spanish born group. Mortality rates were 3.4, 95% CI = (1.4, 7.0) and 8.6, 95% CI = (6.6, 11.0) per 10,000 emergency room visits, respectively.

### Emergency room utilization rates for residents in the city of Lleida

**Figure 1:**
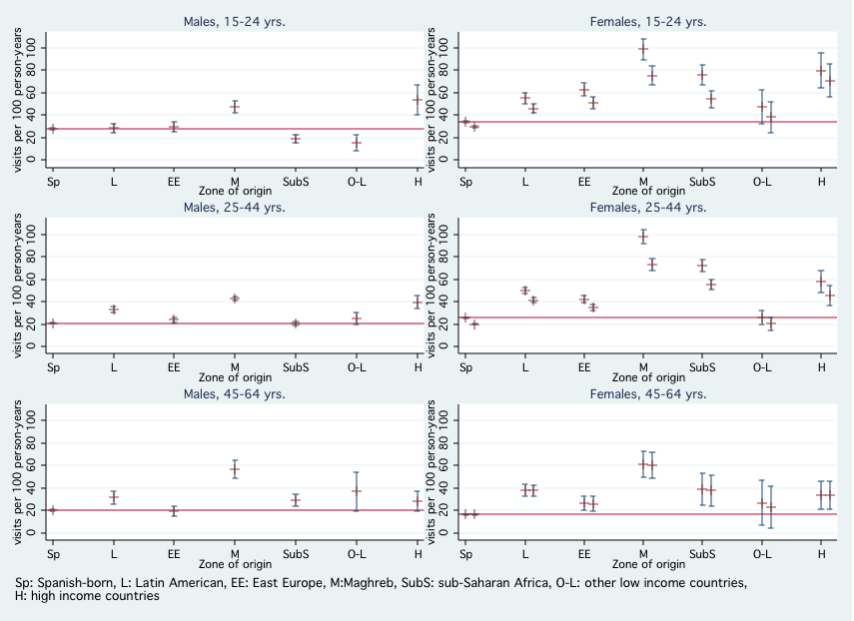
**Utilization rates (visits per 100 person-years) and 95% confidence intervals of Emergency Hospital Services by gender, age and zone of origin**. Arnau de Vilanova Hospital (Lleida, Spain),2004–2005. 1) The horizontal lines indicate the utilization rates of the Spanish-born population. 2) In the females' graphs, there are two values and confidence intervals for each zone of origin. The first indicates the overall utilization rate and the second indicates the utilization rate after excluding gynecologic and obstetric diagnoses.

When estimating utilization rate ratios we observed an interaction between immigrant status and gender. This led us to analyze the data separately by gender. Compared to Spanish born women, immigrant women from Maghreb and sub-Saharan Africa had the highest emergency room utilization rates (overall adjusted RR = 3.5 and 2.6, respectively) (Figure 1 and Table 2). After excluding gynecologic and obstetric diagnoses, utilization rates hardly changed. For both Maghreb and sub-Saharan women, utilization rates were the highest in the 15–24 and 25–44 age groups. Women from Latin America and high-income countries had higher utilization rates than Spanish-born women in all age groups, and Eastern European women used the emergency room more than Spanish-born women in the 15–24 and 25–44 age groups. Women from the Maghreb also had the highest emergency room utilization rates in the 45–64 age group. Rates for women from other low-income countries were not precise due to the low number of visits from this group.

**Table 2:** Age-adjusted rate ratios and 95% confidence intervals (95% CI) for emergency room utilization by country of origin. Arnau de Vilanova Hospital (Lleida, Spain), 2004–2005.

	**Men**		**Women**		**Women (excluding gynecologic and obstetric diagnoses)**	
**Country of origin**	Rate ratio	95% CI	Rate ratio	95% CI	Rate ratio	95% CI

Latin American	1.44	1.35–1.54	1.89	1.81–1.98	1.94	1.85–2.04
Eastern Europe	1.12	1.04–1.22	1.73	1.63–1.83	1.73	1.62–1.85
Maghreb	2.07	1.97–2.16	3.52	3.34–3.71	3.30	3.11–3.50
Sub-Saharan Africa	0.98	0.92–1.04	2.64	2.48–2.81	2.47	2.30–2.65
Other low-income countries	1.09	0.92–1.30	1.15	0.95–1.39	1.12	0.90–1.39
High-income countries	1.83	1.62–2.07	2.27	2.01–2.57	2.32	2.03–2.65

All immigrants (except high-income countries)	1.42	1.38–1.47	2.19	2.13–2.26	2.15	2.08–2.23

Rate ratios and 95% CI were obtained using multivariate Poisson regression models with age as a covariate. Reference country group was Spanish-born.

Compared to Spanish-born patients, male emergency room utilization rates were higher for immigrants from the Maghreb in all age groups (Figure 1 and Table 2). The age-adjusted risk of using the HUAV emergency room was two times higher for men from the Maghreb compared with Spanish born men. The rates were also higher in immigrants from high-income countries, and in Latin American and Eastern European immigrants in the 25–44 group and Latin American and sub-Saharan immigrants in the 45–64 group. Rates were lower for other low-income countries in the 15–24 group.

### Hospital admissions from emergency visits

Emergency room visits by immigrants 15 to 64 years old resulted in hospital admission in 13.4% of cases, 5.5% in males and 20.6% in females, compared with 13.1% in Spanish-born visits, 15.8% in males and 10.1% in females. After excluding the gynecologic and obstetric ER diagnoses, hospital admissions for immigrant and Spanish-born women were similar (10.3% and 9.9%, respectively). Table 3 shows that immigrant males were at lower risk of being admitted to the hospital, as compared with Spanish-born males. Men from Eastern Europe had a similar risk to Spanish-born men. On the other hand, women from all the low-income countries (except Latin America) were at higher risk of being admitted, after adjusting for age. After excluding the visits with gynecologic and obstetric diagnoses, women from other-low income countries, sub-Saharan Africa and the Maghreb, still were at a higher risk of being admitted than their Spanish-born counterparts.

**Table 3:** Odds ratios and 95% confidence intervals (95% CI) of hospital admission from emergency room visits, by country of origin. Arnau de Vilanova Hospital (Lleida, Spain), 2004–2005.

	**Men**		**Women**		**Women (excluding gynecologic and obstetric diagnoses)**	
**Country of origin**	OR	95% CI	OR	95% CI	OR	95% CI

Latin American	0.68	0.54–0.85	0.76	0.68–0.84	0.71	0.60–0.83
East Europe	1.02	0.85–1.22	1.27	1.15–1.41	1.01	0.87–1.18
Maghreb	0.48	0.41–0.56	1.65	1.51–1.81	1.27	1.10–1.46
Sub-Saharan Africa	0.74	0.60–0.90	1.50	1.33–1.70	1.52	1.28–1.82
Other low-income countries	0.32	0.14–0.72	1.94	1.43–2.64	2.24	1.48–3.40
High-income countries	0.75	0.55–1.02	0.69	0.53–0.91	0.77	0.54–1.09

All immigrants (except high-income countries)	0.65	0.59–0.72	1.25	1.18–1.32	1.06	0.97–1.15

OR and 95% CI were obtained using multivariate logistic regression models with age as a covariate. The reference group was Spanish-born.

Hospital diagnoses were similar in men and women in the majority of age groups. A higher percentage of normal delivery was found in immigrant women 15–24 (21.6% of all the hospital admissions from ER), compared to the Spanish-born females (15.3%).

There were 215 hospital deaths in the 15–64 age group, 24 of them in the immigrant group and 191 in the Spanish-born group. Mortality rates were 0.9, 95% CI = (0.6, 1.3) and 2.0, 95% CI = (1.7, 2.3) per 100 hospital admissions, respectively.

## Discussion

The most important finding of this study is that immigrants used EHS more than the Spanish-born population and that there were differences in EHS usage by zone of origin. In the city of Lleida, immigrant women from low-income countries were twice as likely to use EHS than Spanish-born women, after adjusting for age. This value did not change when obstetric and gynecological visits were excluded. Immigrant men use EHS services 42% more than native-born men. Differences in utilization patterns were particularly marked for Maghrebi men and women and sub-Saharan women.

The most common reasons for hospital visits and admissions among immigrants were related to the health needs of the younger populations [16]; the most frequent issues were gynecological-obstetric treatment and general medicine. Upon analyzing the most frequent diagnoses by age groups, we have observed that immigrants and native-born groups are similar. It is possible, however, that there may exist, among the less frequent diagnoses, health issues related to tropical or other diseases endemic in the immigrants' zone of origin that, although representing few cases, may have an important impact on health [17]. With reference to hospital admissions, there is a different pattern in men and women. While male immigrants had a significantly lower risk of hospital admission than Spanish-born men, immigrant women were admitted more frequently than Spanish-born women. When gynecological and obstetric issues were excluded, the risk of hospital admission was the same for immigrant and native-born women.

The results of this study seem to indicate that the utilization profiles of EHS are different by gender. Immigrant men access EHS for health issues that could be treated in a primary care setting, probably influenced by their precarious work situations and inflexible hours. Women immigrants seek EHS more frequently than native-born women, although their health issues are similar in severity to those of Spanish-born women, measured by the proportion of visits that result in hospital admission. It may also be the case that their health issues are less severe, but that language barriers, which are greater among immigrant women than immigrant men, are related to the greater probability of admission.

Among the strengths of this study, the following should be emphasized: first, the fact that the HUAV is the only public hospital in the area makes it possible to obtain comprehensive utilization rates by zone of origin, something that has not been possible in previously published Spanish studies. Second, this is a study that provides information on the use of EHS during an important transition period for the demographic composition of the Spanish society. Third, this is a broad study that examines all EHS activity in a community hospital over a period of two years. Fourth, the area where the study was carried out has an important immigrant population (17%), nearly double that of Spain during the same time period.

Among the limitations of the study are the following: first, the study was restricted to patients between the ages of 15 and 64, in order to increase comparability between immigrant and Spanish-born groups. As we mentioned in the methods section, the hospital data did not include information on parents' zone of origin for children born in Spain and, on the other hand, immigrants older than 64 were a very small group compared to Spanish-born patients in this age group. Second, there may be bias in the estimation of utilization rates due to the use of EHS by immigrants not included in the official city census – in some cases, foreigners who were visiting the city or, in other cases immigrants living in the city of Lleida not registered in the census. Third, close to 10% of the diagnoses by EHS were not correctly coded, and 18% were non-specific diagnoses. However, these coding errors or unspecified diagnosis occurred with the same frequency in both immigrant and native-born patients. Forth, EHS usage in private clinics in Lleida city has not been included in the estimation of utilization rates. Since some percentage of the population, especially Spanish-born patients, may have sought care in the private sector, the differences in usage rates between immigrants and native-born found in this study may be smaller [18]. According to the Health Department, the percentage of the population that seeks EHS in private clinics is less than 5%. Rodriguez et al studied the utilization of health services in Spain by gender, type of insurance access and level of education [19]. They showed that having private access has no impact on hospitalizations and emergency room visits, but increases the probability of visits to specialists and to private doctors. Immigrant women probably use the EHS for gynecological and obstetric care that Spanish-born women receive from private health services or in the public primary care follow-up programs. Fifth, we did not have data on health status of the immigrants by region of origin. So far, health surveys in Catalonia or Spain have not had samples large enough to obtain representative data on health status by zone of origin. Sixth, our study was based on visits, not on patients. Since visits from the same patient are not independent and this fact may produce overdispersion in a Poisson regression model, we also estimated the utilization rate ratios with a negative binomial model and we obtained very similar estimates for the rate ratios and p-values, meaning that our Poisson estimated rate ratios are robust to repeated events. Finally, another important limitation is that we did not collect information on variables such as, socioeconomic status, legal status, or time in Spain, which may be related to health status and to health services utilization. In Santander (Spain) Braun *et al *observed that patients with lower socioeconomic status had more repeated visits to the EHS [20]. This finding can be explained, according to the authors, by worse health status or inadequate use of services – using primary care services less frequently.

Of the total number of EHS visits to our hospital in the years 2004 and 2005, 16% were by immigrants. This percentage was 15.6% when only immigrants from low-income countries were considered. These data are similar to those reported by Cots *et al *in the Hospital del Mar in Barcelona during the years 2002 and 2003 where 15.5% of EHS visits were by immigrants from low-income countries [10]. This study also emphasizes obstetric issues as among the principal causes for seeking EHS services in immigrant women.

When Cots *et al *analyzed the impact of immigration on the cost of emergency visits in a Barcelona hospital, they found that immigrants tend to use the EHS in preference to other health services like primary or specialized care, but health care expenditures for immigrants were lower than those for Spanish born persons [10,13]. As Cots *et al *mention, the over utilization of hospital emergency services involves the use of expensive high-intensity resources to respond to non-urgent conditions that could be managed in the primary care setting. A limitation of the Cots' study is the fact that they could not estimate EHS utilization rates due to the existence of other public hospitals in the city of Barcelona. Carrasco-Garrido *et al *in 2003, based on a immigrant/native-born matched subsample of the Spanish National Health Survey, did not find significant differences in the percentages of people that had at least one emergency visit during the last year, and reported a higher percentage of individuals hospitalized during the last 12 months among immigrants than in the Spanish-born population [11]. A limitation of the Carrasco-Garrido study was that the immigrant study sample was small (500 individuals) and they did not get information on the number of EHS visits. Cacciani *et al *found that immigrants in Italy used fewer health resources than the resident population, with a ratio of 0.8 for acute care and 0.7 for ambulatory care [21]. In their study the immigrants had a higher rate of hospitalization than the resident population for some specific causes such as injuries, infectious diseases, deliveries, induced abortions and ill-defined conditions.

As other studies have shown, recent immigrants face several barriers to the use of health services [22,23]. Because the Spanish health system is universal for individuals registered in a municipality of residence, almost all immigrants have access to health care. Nevertheless, there may be a small fraction of immigrants that have access only to EHS. Other barriers to the use of regular health care that have been described are: lack of knowledge of the health system, particularly primary care services; language and cultural differences; socioeconomic status; and precarious employment situations which make it impossible for patients to leave work to go to the doctor during normal hours. These jobs are frequently precarious, have long working hours, employers can fire workers at will and the work situation is often extra-legal. In a study in Navarra (Spain), Parra *et al *estimated that, among working immigrants, three out of ten men and five out of ten women were illegaly employed [24]. Aranaz *et al*, in a 2002 study in Alicante (Spain), observed that approximately half of the immigrant population was unaware of how primary care services function [25]. Norredam *et al *compared patients of Danish origin with immigrants, and found that immigrants have more irrelevant EHS claims and reported going to the EHS because they could not contact a general practitioner [26]. Another study from Norredam *et al *in Copenhagen compared the emergency room utilization rates of immigrants and Danish-born residents and found higher utilization rates for persons born in Somalia, Turkey, and the former Yugoslavia [27]. They mention lack of knowledge about the Danish healthcare system, as well as barriers to seeking primary care including language, fear of discrimination, and low satisfaction with primary care as possible causes of the EHS over-utilization. Leclere *et al *in the US found that language is one of the primary barriers faced by immigrants seeking medical attention [22]. Another important finding of Leclere *et al *was that recent immigrants, regardless of their insurance status, face barriers to care that are similar to the uninsured. Guendelman *et al *studied access to care for immigrant children in poor working families in California [28]. They found that insured immigrant children fared worse than insured nonimmigrant on almost every indicator of access and use. They suggest that one reason for the disparities among the insured may be that not all insurance is alike. The disparities that placed immigrant children at a disadvantage were: lacking a usual care source, postponing or forgoing dental or medical care, having fewer physician visits, and avoiding the emergency room. Guendelman *et al *also concluded that non-financial barriers may exert an important effect on access for immigrants.

## Conclusion

In summary, these results seem to suggest that in Lleida (Spain) immigrants use more EHS than the Spanish-born population. Future research should explore whether the same pattern is found in other areas of Spain, and whether EHS use is attributable to health needs, barriers to access to the primary care services or similarities in the way that immigrants access health care in their countries of origin.

## Competing interests

The authors declare that they have no competing interests.

## Authors' contributions

MR participated in the design and the coordination of the study, performed the statistical analysis and helped to draft the manuscript. XC, JSG, MA and MCS participated in the design of the study, interpreted the results and helped to draft the manuscript. AB participated in the statistical analysis and helped to draft the results. All authors read and approved the final manuscript.

## Pre-publication history

The pre-publication history for this paper can be accessed here:


